# A Lucky Man

**Published:** 1875-12

**Authors:** 


A Lucky Man.—Twenty clerks in a store, twenty hands in a printing office, twenty apprentices in a shipyard, twenty young men in a village—all want to get along in the world and expect to do so. One of the clerks will become a partner, and make a fortune; one of the compositors will own a newspaper, and become an influential citizen; one of the apprentices will become a master builder; one of the young villagers will get a handsome farm, and live like a patriarch—but which one is the lucky individual! Lucky? There is no luck about it. The thing is almost as certain as the rule of three. The young fellow who will distance his competitors is he who masters his business, who preserves his integrity, who lives cleanly and purely, who devotes his leisure to the acquisition of knowledge, who gains friends by deserving them and who saves his spare mouey. There are some ways to fortune shorter than this old, dusty highway; but the staunch men of the community, the men who achieve something worth having, good fortune good name, and serene old age, all go in this hard, dirty road.

---

Flowers as Time Keepers—Linnæus, a great student of nature once made what he called a floral clock. It consisted of three divisions; first, those flowers that by their opening or closing foretold the weather; second, those that opened at sunrise and closed at sunset; and lastly, flowers that opened and closed at fixed and invariable hours. He had a flower representing every hour of the day and night, and these were always exact in telling the hour. The African marigold opens constantly at seven o'clock and remains open until four, if the weather be fair. If it does not open or if it closes before that hour, it is certain that rain will fall during the day.

### MY LITTLE BROTHER.

BY ALICE CAREY.

Of all the beautiful pictures
That hang on memory's wall,
Is one of a dim, old forest,
That seemeth best of all;
Not for its gnarled oaks olden,
Dark with the misletoe;
Not for the violets golden,
That sprinkle the vale below;
Not for the milk-white lilies
That lean from the fragrant hedge,
Coquetting all day with the sunbeams,
And stealing their golden edge;
Not for the vines on the upland,
Where the bright, red berries rest;
Nor the pinks, nor the pale, sweet cowslip'
It seemeth to me the best.

I once had a little brother
With eyes that were dark and deep—
In the lap of that olden forest
He lieth in peace asleep.
Light as the down of the thistle,
Free as the winds that blow,
We roved there the beautiful summers—
The summers of long ago;
But his feet on the hills grew weary,
And one of the autumn eves
I made my little brother
A bed of the yellow leaves.

Sweetly his pale arms folded
My neck in meek embrace,
As the light of immortal beauty
Silently covered his face;
And when the arrows of sunset
Lodged in the tree tops bright,
He fell in his saintly-beauty,
Asleep by the gates of light.
Therefore, of all the pictures
That hang on menory's wall,
The one of the dim old forest
Seemeth the best of all.

